# Privacy Preserving Multi-Party Key Exchange Protocol for Wireless Mesh Networks

**DOI:** 10.3390/s22051958

**Published:** 2022-03-02

**Authors:** Amit Kumar Roy, Keshab Nath, Gautam Srivastava, Thippa Reddy Gadekallu, Jerry Chun-Wei Lin

**Affiliations:** 1 Department of Computer Science and Engineering, National Institute of Technology Mizoram, Aizawl 796012, India; amitroy.cse@nitmz.ac.in; 2 Department of Computer Science and Engineering, Indian Institute of Information Technology, Kottayam 686635, India; keshabnath@iiitkottayam.ac.in; 3 Department of Mathematics and Computer Science, Brandon University, Brandon, MB R7A 6A9, Canada; srivastavag@brandonu.ca; 4 Research Centre for Interneural Computing, China Medical University, Taichung 40402, Taiwan; 5 School of Information Technology, Vellore Institute of Technology, Vellore 632014, India; thippareddy.g@vit.ac.in; 6 Department of Computer Science, Electrical Engineering and Mathematical Sciences, Western Norway University of Applied Sciences, 5020 Bergen, Norway

**Keywords:** handover, authentication, privacy, tickets, computation cost, communication cost

## Abstract

Presently, lightweight devices such as mobile phones, notepads, and laptops are widely used to access the Internet throughout the world; however, a problem of privacy preservation and authentication delay occurs during handover operation when these devices change their position from a home mesh access point (HMAP) to a foreign mesh access point (FMAP). Authentication during handover is mostly performed through ticket-based techniques, which permit the user to authenticate itself to the foreign mesh access point; therefore, a secure communication method should be formed between the mesh entities to exchange the tickets. In two existing protocols, this ticket was not secured at all and exchanged in a plaintext format. We propose a protocol for handover authentication with privacy preservation of the transfer ticket via the Diffie–Hellman method. Through experimental results, our proposed protocol achieves privacy preservation with minimum authentication delay during handover operation.

## 1. Introduction

**Figure 1:**
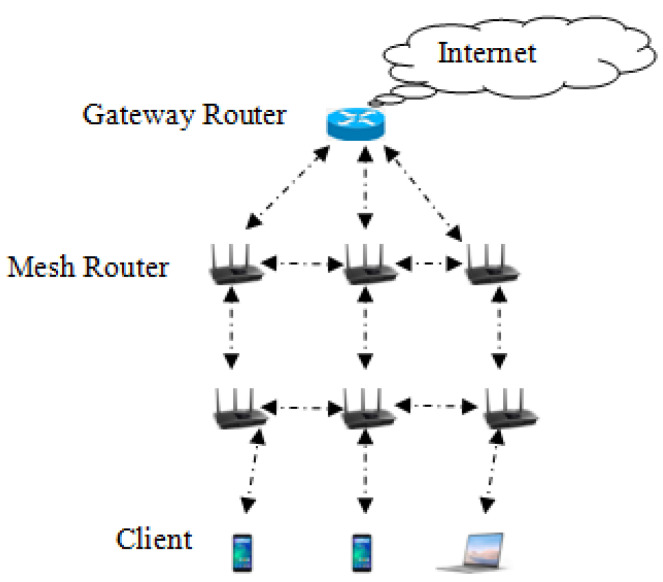
Architecture of WMN.

As compared to conventional networks such as LAN and MANET, wireless mesh networks (WMN) have become the most promising network presently due to their advanced features. Due to their capacity to be self-organized and self-healing, WMN are the most favorable network [1]. Advanced features of WMN allow continuous network access to the end-users. Three mesh entities, namely gateway routers (GW), mesh routers (MR), and mesh clients (MC) form the architecture of WMN as shown in Figure 1. Mesh routers are also called mesh access points (MAP), which forward the mesh client’s request to the gateway router (GW) for Internet access [2,3,4,5]. Due to the non-static nature, mesh clients can change their position from a home mesh access point to a foreign mesh access point. As a result, a secure handover authentication process should be carried out among mesh entities. Successful authentication during handover permits the client to join and access the Internet under a foreign MAP [6,7,8]. In the past, numerous protocols were proposed for handover authentication that are based on tickets [9,10]; however, they came up with certain issues and limitations, which are discussed in Section 2. The proposed multi-party key exchange protocol presented in this paper offers the privacy of the transfer ticket. Our proposed multi-party key exchange protocol is an extension of the Diffie–Hellman protocol [11]. The privacy of a transfer ticket is preserved throughout the login authentication process (LAP) and handover authentication process (HAP) among the mesh entities. The protocol does not require any MAC key generation and master key generated from the AS, as it was required in the existing protocols. Mostly in existing protocols, the transfer ticket is issued by the authentication server (AS), but in our proposed work, it is issued by the mesh access point (MAP) within one hop; therefore, the presence of the AS is not considered in our proposed protocol throughout the handover process.

### Discussion and Contribution

In this paper, we present an efficient authentication protocol during handover operation along with privacy preservation of tickets shared over the insecure channel. Our proposed protocol is analyzed against the Li et al. protocol because in this protocol authentication is carried out via tickets and computations are performed mostly by the TA and MAP [12]; secondly, the amount of communication cost is minimized (i.e., three-way handshake performed) during the handover authentication process; lastly, involvement of the third party during handover operation is omitted. All these properties make the protocol to be lightweight for mobile users in WMN; however, we analyzed the existing protocol in detail in Section 3 and found certain drawbacks. In this paper, our main contributions can be described as follows:

- We propose a multi-party key exchange protocol to generate a common secret key (CSK), which is shared among the MAP within a group. This common key is used for encrypting and decrypting the transfer tickets shared during the handover operation and offers privacy to the transfer tickets.
- We consider only symmetric key-based operations during the handover operation, which results in minimal computational cost.
- We achieve a complete handover authentication process with minimal communication cost, i.e., one-way handshake, which is efficient compared to two-way and three-way handshake of existing protocols.

The remainder of the paper is as follows: Section 2 describes the related works. In Section 3, the analysis of existing work and its drawbacks are discussed in detail. The proposed multi-party key exchange protocol and Diffie–Hellman protocol are discussed in Section 4. Proposed protocols during LAP and HAP are discussed in Section 5. Section 6 describes the experimental results and Section 7 concludes the paper.

## 2. Related Work

In this section, we discuss some of the existing protocols related to our proposed protocol. The existing protocols were mainly concerned with the handover authentication process carried out through a ticket-based approach.

Kassab et al. [13] proposed a secure protocol for proactive authentication for the IEEE 802.11F standard network during handover. During the handover process, the client sends a request message to the foreign access point to join the network. On acceptance, the foreign access point sends the message to the authentication server. The authentication server verifies the message. On successful verification, the authentication server issues an acceptance message to the foreign access point, which allows the client to join the network under the foreign access point; however, certain limitations were found in the protocol during the handover process. Limitations such as authentication delay due to verification of the request message by AS were required in a multi-hop fashion. Li et al. [14] proposed a handover protocol where re-authentication is strongly considered for the IEEE 802.11i standard network. Firstly, for mutual authentication, the complete process of authentication was formed among the mobile station and AS. Secondly, the AS issued a list of handover tickets of the neighboring access point to the mobile station. These lists of handover tickets allowed the mobile station to re-authenticate itself to the neighboring AP’s during the handover operation; however, storing this list of tickets consumed massive storage space at mobile stations, which are usually resource constrained. Li et al. [15] proposed a protocol during handover based on broadcast authentication. The protocol allowed the client to be authenticated by the authentication server. During the handover operation, the authentication server issued and broadcast the tickets to each mesh access point, which allowed the clients to authenticate during the handover process; however, a massive authentication delay occurs due to multi-hop authentication required from the authentication server. He et al. [16] proposed a handover authentication protocol with a two-way handshake to complete the handover authentication process. The protocol was based on pre-shared pseudo identities (PIDi) generated by the AS to the mesh clients. However, a pseudo-identity involves the bilinear pairing operation, which results in high computational cost. Moreover, this approach pre-shared pseudo identities (PIDi) to the clients, putting extra load on clients’ constrained resources. Xu et al. [17] proposed a protocol for wireless mesh network during handover authentication. The protocol allowed the authentication server (AS) to pre-distribute the tickets to the clients. These tickets were used during the re-authentication process. The client forwards the ticket to the intended mesh router based on its identity. Later, the mesh router verified the ticket sent by the client and on successful verification the client is re-authenticated; however, storing these pre-distributed tickets consumed massive storage space on the client side, which is resource constrained. Rathee et al. [18] proposed a secure protocol for WMN during handover operation. The protocol generates two keys, namely, the master key and group key shared between the authentication server (AS), mesh router, and mesh clients to authenticate each other. Then, the AS issued the ticket to the client and mesh router to authenticate each other during the handover process; however, the protocol comes up with certain limitations. First, during the handoff phase, target FMAP verifies the MC by comparing the tickets in step 2 but the protocol lacks the ability to verify the target FMAP by the MC side. Second, without verifying the target FMAP, the temporary session key is generated by both sides in step 3. Overall the protocol performs a 3-way handshake without completing the authentication process from the MC side. Third, a massive message was exchanged which leads to high communication costs during handover operation. Second, messages were exchanged in a plaintext format over the insecure channel, which violates the integrity of the message easily. Fourth, AS verifies the ticket and the client in a multi-hop fashion that leads to authentication delay. Wang et al. [19] proposed a batch handover authentication protocol based on the pre-distribution of handover keys to minimizing the authentication delay. The protocol preserved the privacy of the client where the identity of the foreign mesh router (MRj) and timestamp of the client (TMCi) was unknown to the attacker; however, storing these pre-distributed tickets consumed massive storage space at the client side, which are resource-constrained. Rekik et al. [20] proposed an optimized, secure authentication protocol based on extensible authentication protocol (EAP) for handover authentication; however, the protocol requires multi-hop authentication from the AS, which results in an authentication delay.

To improve the handover authentication process, privacy was considered in Tsai et al. [21] protocol, Fu et al. [22] protocol, and Zhu et al. [23] protocol. These protocols preserved the privacy of the clients with a three-way handshake to complete the handover authentication process; however, to complete the three-way handshake protocol, it suffered from high computational cost. Yang et al. [24] proposed an efficient handover authentication protocol with a two-way handshake to complete the handover authentication process. The protocol was based on the group signature performed by the group manager (mesh access point). The roaming client is required to forward the group signature to the foreign mesh access point (FMAP) to validate its authentication; however, the protocol was based on bilinear pairing, which results in high computational cost. Table 1 compares the existing protocols with different parameters during handover operation.

## 3. Analysis of Existing Protocol

In this section, we investigate in detail the existing protocol proposed by Li et al. [12] and discuss the security threat present in the protocol. The protocol considered a trust model, which employed a ticket agent (*TA*). The *TA* issues the MAP ticket and user ticket to authenticate each other during the login process and handover process. In the mesh network, *TA* acts as a centralized authority. The following shows the various faiths built among the mesh entities.

- **TA-MAP**: On a request of MAP ticket, faith is built between TA and the MAP.
- **TA-user**: On a request of user ticket, faith is built between TA and the user.
- **MAP-user**: Through MAP ticket and user ticket, faith is built between MAP and the user.
- **MAP_1_-MAP_2_**: Among neighboring MAPs, faith is built through their public key certificate. Faith among neighboring MAP allows the user to connect to any neighboring MAP.

### 3.1. Types of Ticket Issued to MAP and User for Mutual Authentication

- **User tickets (*T_C_*)**: Faith between user and MAP is built through user ticket. The legality of user is proved to MAP through *TC*. *TC* contains the following elements
  (1) $$T_{C}=\left.\{I_{C},I_{A},\tau_{exp},P_{C},Sig_{A}\right.\}$$
  where,
  $I_{C}$ = User identity.
  $I_{A}$ = TA identity.
  $\tau_{exp}$ = expiry time of $T_{C}$.
  $P_{C}$ = User’s public key.
  $Sig_{A}$ = TA digital signature.
- **MAP ticket ($T_{M}$)**: Builds faith between MAP and User. The legality of MAP is proved to user through $T_{M}$. $T_{M}$ contains the following elements
  (2) $$T_{M}=\left.\{I_{M},I_{A},\tau_{exp},P_{M},Sig_{A}\right.\}$$
  where,
  $I_{M}$ = MAP identity.
  $I_{A}$ = TA identity.
  $\tau_{exp}$ = expiry time of $T_{M}$.
  $P_{M}$ = MAP’s public key.
  $Sig_{A}$ = TA digital signature.
- **Transfer tickets ($\Theta_{C}$)**: Builds faith between user and FMAP (e.g., $MAP_{2}$). After, the mutual trust/faith is built between user and home MAP, $\Theta_{C}$ is generated by a home MAP (e.g., $MAP_{1}$). User proved its legality to $MAP_{2}$ through $\Theta_{C}$. $\Theta_{C}$ contains the following elements
  (3) $$\Theta_{C}=\left.\{I_{C},I_{M},I_{A},\tau_{exp},V_{K_{MAC}}\left(I_{C},I_{M},I_{A},\tau_{exp}\right)\right.\}$$
  where,
  $I_{C}$ = User identity owning $\Theta_{C}$.
  $I_{M}$ = MAP identity issuing $\Theta_{C}$.
  $I_{A}$ = TA identity.
  $\tau_{exp}$ = expiry time of $\Theta_{C}$.

### 3.2. The Login Authentication Protocol (LAP)

Assume that the trust agent (TA) issued a user ticket ($T_{C}$) to user *C* and MAP ticket ($T_{M}$) to $MAP_{1}$. Now the user and $MAP_{1}$ exchanged the tickets for mutual authentication. Steps for exchanging the tickets for mutual authentication are as follows:

(4) $$C\to MAP_{1}:I_{C}$$

(5) $$MAP_{1}\to C:T_{M1}$$

(6) $$C\to MAP_{1}:E_{P_{M1}}\left(T_{C},N_{C1},N_{C2}\right)$$

(7) $$MAP_{1}\to C:E_{P_{C}}\left(N_{M1},N_{M2}\right)$$

(8) $$C\to MAP_{1}:N_{M2}$$

(9) $$MAP_{1}\to C:N_{C2},\left(\Theta_{C}\right)$$

**Step 1:** For Internet access, the identity ($I_{C}$) of *C* is broadcast as a request message to $MAP_{1}$.
**Step 2:** On the acceptance of the request message, $MAP_{1}$ send its ticket ($T_{M1}$) to user *C*. After receiving $T_{M1}$ by *C*, $T_{M1}$ is verified through signature ($Sig_{A}$) and through expiry time $\tau_{exp}$ exists in $T_{M1}$.
**Step 3:** If verification of $T_{M1}$ is successful, then the public key $P_{M1}$ of $MAP_{1}$ is extracted from $T_{M1}$ by *C*. Then User *C* encrypts the ticket $T_{C}$, nonces $N_{C1}$, and $N_{C2}$ by using the public key $P_{M1}$ and sends to $MAP_{1}$. On acceptance of the message, $MAP_{1}$ decrypts the message with its private key and verifies the ticket $T_{C}$. Verification is achieved through signature ($Sig_{A}$) and expiry time $\tau_{exp}$ present in $T_{C}$. $MAP_{1}$ ignores the ticket $T_{C}$, if the verification fails.
**Step 4:** After verification is successful, public key $P_{C}$ of *C* is extracted from $T_{C}$ by $MAP_{1}$. Later, $MAP_{1}$ encrypts two nonce $N_{M1}$ and $N_{M2}$ using $P_{C}$ and forwards the encrypted message to user. Meanwhile, $MAP_{1}$ compute its shared MAC key $K_{MAC}$ = $N_{C1}$‖$N_{M1}$ and pairwise master key $PMK_{0}$ = $N_{C1}$‖$N_{M1}$. On the acceptance of an encrypted message, this message is decrypted by user *C* with its own private key to gain $N_{M1}$ and $N_{M2}$. Later, user *C* computes its shared MAC key $K_{MAC}$ = $N_{C1}$‖$N_{M1}$ and pairwise master key $PMK_{0}$ = $N_{C1}$‖$N_{M1}$. The nonces $N_{C1}$, $N_{C2}$, $N_{M1}$, and $N_{M2}$ are secured through asymmetric cryptography.
**Step 5:** After calculating a shared MAC key and pairwise master key, user *C* sends the nonce $N_{M2}$ to $MAP_{1}$. On the acceptance of a nonce $N_{M2}$, $MAP_{1}$ verifies a nonce $N_{M2}$ with a nonce issued by $MAP_{1}$ itself earlier in Equation (7). $MAP_{1}$ ignores the nonce, if $N_{M2}$ does not match with the earlier nonce.
**Step 6:** After successful verification till step 5, $MAP_{1}$ generates a transfer ticket $\Theta_{C}$. Then, $MAP_{1}$ sends to user *C* the nonce $N_{C2}$ and transfer ticket $\Theta_{C}$. User *C* after receiving the $N_{C2}$ and $\Theta_{C}$, verifies the nonce $N_{C2}$ by checking with the nonce issued earlier by *C* itself in Equation (6). User *C* ignores the message if the $N_{C2}$ does not match. Finally, step 1 to step 6 concludes the login authentication protocol. Later, $\Theta_{C}$ allows the user *C* to initiate the handover authentication process from home $MAP_{1}$ to foreign MAP.

### 3.3. The Handover Authentication Protocol (HAP)

To initiate an efficient handover operation, $MAP_{1}$ pre-distributes the shared keys to all its neighboring MAP. These keys are shared between the user and $MAP_{1}$ during the login authentication process. It is assumed that all the MAP contain its neighboring MAP public key certificates. On successful completion of the login authentication process, $MAP_{1}$ pre-distributes the encrypted shared keys, which includes $I_{C}$, $I_{M1}$, key $K_{MAC}$, and pairwise master key $PMK_{0}$ to its neighboring $MAP_{x}$. The encryption is performed via public key $P_{x}$ of neighboring $MAP_{x}$. After receiving the encrypted shared keys, $MAP_{x}$ uses its private key to decrypt it. Finally, the new authentication process is carried out with user *C* through these shared keys. During the handover process from $MAP_{1}$ to $MAP_{x}$, user *C* performs the following steps:

(10) $$C\to MAP_{x}:\Theta_{C},N_{C},V_{K_{MAC}}\left(N_{C}\right)$$

(11) $$MAP_{x}\to C:N_{M},V_{K_{MAC}}\left(N_{C},N_{M}\right)$$

(12) $$C\to MAP_{x}:N_{M},V_{K_{MAC}}\left(N_{M}\right)$$

**Step 1:** User *C* sends $\Theta_{C}$, new nonce $N_{C}$ and MAC $V_{K_{MAC}}$ ($N_{C}$) to foreign $MAP_{x}$ shown in Equation (10). On the acceptance of the message, $MAP_{x}$ verifies the accuracy of $V_{K_{MAC}}$ ($N_{C}$) by using previously received $K_{MAC}$ from the home $MAP_{1}$. If the verification is successful, $MAP_{1}$ checks the elements in $\Theta_{C}$ to verify the legality of $\Theta_{C}$. Likewise, only user *C* with $K_{MAC}$ knowledge could generate a valid pair of ($N_{C}$, $V_{K_{MAC}}$ ($N_{C}$)).
**Step 2:** If the validation of $\Theta_{C}$ is successful, $MAP_{x}$ send a nonce $N_{M}$ and $V_{K_{MAC}}$ ($N_{C}$, $N_{M}$) to user *C* shown in Equation (11).
**Step 3:** On the acceptance of a message, user *C* sends $N_{M}$ and $V_{K_{MAC}}$ ($N_{M}$) to $MAP_{x}$ shown in Equation (12). On the acceptance of $N_{M}$ and $V_{K_{MAC}}$ ($N_{M}$), $MAP_{x}$ verifies the $V_{K_{MAC}}$ ($N_{M}$). On successful verification, the user’s identity is approved as legal and concludes the HAP.

**Figure 2:**
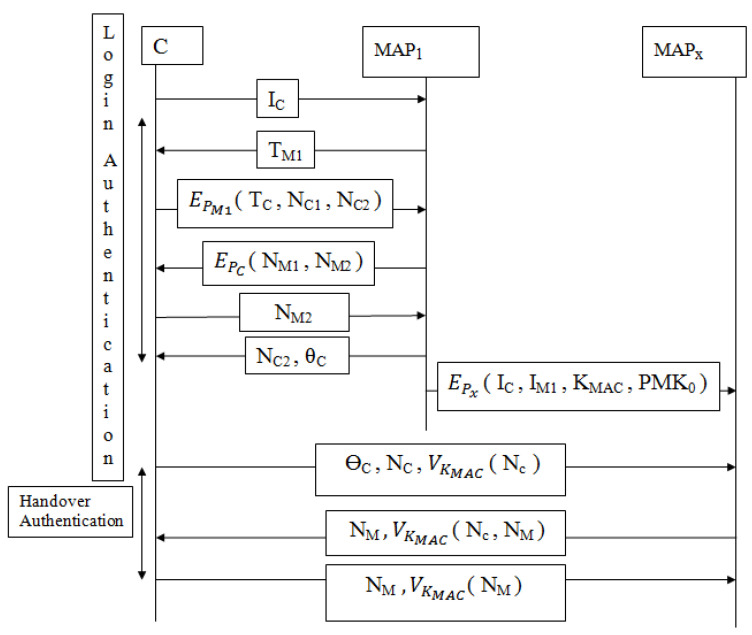
Li et al. [12] protocol during LAP and HAP.

**Discussion**: We analyze the Li et al. [12] protocol in detail and found certain limitations and security threats in the protocol, which are highlighted below:

Two different authentication protocols are considered in the existing protocol: 1. To initiate mutual authentication, login authentication protocol (LAP) is considered. 2. To initiate the handover process, handover authentication protocol (HAP) is considered as shown in Figure 2. Both LAP and HAP rely on certain keys such as pairwise master key and group transient key for authentication between users and MAP. Within the network, users are offered constraint power; therefore, the exchange of these keys should be minimized. Both LAP and HAP protocols suffered from security threats. Firstly, throughout LAP the information $T_{M1}$, $N_{M2}$, $N_{C2}$ and $\Theta_{C}$ are shared in a plaintext format as $MAP_{1}$→*C*: $T_{M1}$ shown in Equation (5), *C*→$MAP_{1}$: $N_{M2}$ as shown in Equation (8) and $MAP_{1}$→*C*: $N_{C2}$, $\Theta_{C}$ as shown in Equation (9). As a result, an intruder could easily acquire this information and misuse it.

Secondly, $\Theta_{C}$ are shared in the plaintext format as C→$MAP_{x}$: $\Theta_{C}$, $N_{C2}$, $V_{K_{MAC}}$ ($N_{C}$) during HAP as shown in Equation (10). As a result, an intruder could easily tamper the elements of $\Theta_{C}$ such as $I_{C}$, $I_{M}$, $I_{A}$, $\tau_{exp}$ and violates the integrity of transfer ticket ($\Theta_{C}$); therefore, an intruder could easily eavesdrop on these exchanged messages at the time of the authentication process. Further, the intruder could replay these messages and try to obtain successful authentication as a user to access the network.

## 4. Proposed Multi-Party Key Exchange Protocol

The proposed multi-party key exchange protocol is an extension of the Diffie–Hellman approach, which is performed within a group by the ticket agent (TA) and the MAP, where the ticket agent (TA) is known as a group controller (GC). The ticket agent (TA) generates the common secret key (CSK) and shares it in an encrypted form among neighboring MAP. Further, the CSK is employed for encryption and decryption of the transfer ticket during LAP and HAP between MAP and users. The detailed procedure for multi-party key exchange protocol is presented in Algorithm 1.
**Algorithm 1:** Muti-party key exchange algorithm.
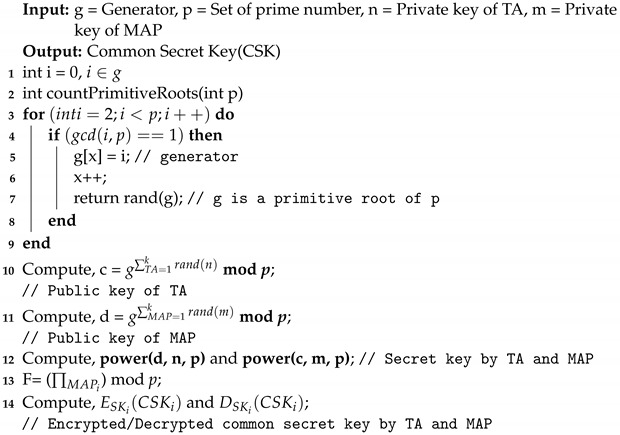


In Algorithm 1, line 3 to line 7 returns the primitive roots less than the modulo prime *p* and the value is stored in an array g[]. In line 10 of Algorithm 1, the public keys for the $i^{th}$ TA is computed as $TA_{i}=g^{n_{i}}$ mod *p*. In line 11 of Algorithm 1, the public keys for the $i^{th}$ MAP is computed as $MAP_{i}=g^{m_{i}}\;\mathrm{mod}\;p$. After the generation of public keys by $TA_{i}$ and $MAP_{i}$, both parties exchanged their public keys. In line 12 of Algorithm 1, the secret keys are computed by both the parties, where the value of n and m are chosen randomly. $TA_{i}$ computes the secret key as $SK_{i}=MAP_{i}^{n_{i}}\;\mathrm{mod}\;p$ and $MAP_{i}$ computes the secret key as $SK_{i}=TA_{i}^{m_{i}}\;\mathrm{mod}\;p$. Both parties generates the same secret keys in line 12. This keys are further used for encrypting and decrypting the common secret key (CSK) as shown in line 14. In line 13, the common secret key generated by the $TA_{i}$ is computed as $CSK_{i}=\left(\prod_{MAP_{i}}\right)$ mod *p*. TA generates the common secret key (CSK) by adding all the public keys received from each MAP’s using product of sum operation (∏). Later, $CSK_{i}$ is used for encrypting and decrypting the transfer ticket throughout the LAP and HAP.

### Reason to Considered Diffie–Hellman Key Exchange Protocol

We considered an extension of the Diffie–Hellman protocol [11] in our proposed protocol, which allows multiple users to securely exchange the keys over an insecure channel. Further, the keys are used for encrypting and decrypting the message. The difficulty and complexity of discrete logarithms to compute directly reflect the advantage of the Diffie–Hellman algorithm. The difficulty and complexity to crack the Diffie–Hellman protocol can be discussed as follows

- Discrete logarithms can be defined as a primitive root that belongs to the prime number *p* whose powers modulo *p* produce 1 to p1 integers; therefore, if *a* consider as prime number *p*, then $a^{1}\;\mathrm{mod}\;p,a^{2}\;\mathrm{mod}\;p,\ldots ,a^{p1}\;\mathrm{mod}\;p$ are distinct and contain integers from 1 to p1 in some permutation.
- Discrete Logarithm Problem: It is considered as a multiplicative cyclic group. Where G=(g) is the generator of the cyclic group with element *h* of *G*; therefore, search unique integer *x*, where $g^{x}=h$, and *x* is the discrete logarithm of *h* with base *g*.
- Computational Diffie–Hellman Problem (CDH): It is defined as a cyclic group (G) with generator *g* and $g^{x1},g^{x2}\in G$; therefore, known values are y1 = $g^{x1}$ and y2 = $g^{x2}$ whereas x1 and x2 are unknown, hence search $y=g^{x1}g^{x2}$. CDH assumption is considered in most of the security of the cryptosystem. CDH assumption is associated with discrete logarithm assumption, where computing the discrete logarithm for value base a generator *g* is hard.

## 5. Proposed Protocol during Login Authentication Protocol (LAP) and Handover Authentication Protocol (HAP)

To overcome the limitations present in [12], we proposed a multi-party key exchanged protocol shown in Section 4. We consider the existing ticket types in our proposed protocol with a change in transfer ticket $\Theta_{C}$ elements. Changed elements in $\Theta_{C}$ are given as

(13) $$\Theta_{C}=\left.\{I_{C},I_{M},I_{A},N_{i},\tau_{exp}\right.\}$$

where,

$I_{C}$ = Identity of the user owning $\Theta_{C}$.

$I_{M}$ = Identity of the MAP issuing $\Theta_{C}$.

$I_{A}$ = TA Identity.

$\tau_{exp}$ = $\Theta_{C}$’s expiry time.

$N_{i}$ = nonce to prevent replay attack.

### 5.1. Proposed Protocol for Login Authentication Protocol (LAP)

Initially, the user ticket and the MAP ticket were issued by the TA. Both $MAP_{1}$ and user exchanged their tickets for mutual authentication. Order of tickets exchanged between $MAP_{1}$ and User are as follows.

(14) $$C\to MAP_{1}:\left(I_{C},P_{C}\right)$$

(15) $$MAP_{1}\to C:E_{P_{C}}\left(T_{M1},N_{M1}\right)$$

(16) $$C\to MAP_{1}:E_{P_{M1}}\left(T_{C},N_{M1}\right)$$

(17) $$MAP_{1}\to C,FMAP:E_{CSK_{i}}\left(\Theta_{C}\right)$$

**Step 1:** Identity and public key of user *C* is broadcast as a request message to $MAP_{1}$ to allow Internet access in Equation (14).
**Step 2:** After the message received, $MAP_{1}$ extracts the users public key $P_{C}$. $MAP_{1}$ uses the public key to encrypt the ticket $T_{M1}$ and a nonce $N_{M1}$ and sends to user *C* in Equation (15). On the acceptance of encrypted $T_{M1}$ and a nonce $N_{M1}$, the user decrypts it by using its private key. After decryption, the user verifies a $T_{M1}$ through $Sig_{A}$ and $\tau_{exp}$ that resides within $T_{M1}$.
**Step 3:** After successful verification of $T_{M1}$, the public key $P_{M1}$ of $MAP_{1}$ is extracted by the user from $T_{M1}$. The user encrypts $T_{C}$ and nonce $N_{M1}$ using $P_{M1}$ and send towards $MAP_{1}$ in Equation (16). On the arrival of $E_{P_{M1}}$ ($T_{C}$, $N_{M1}$), $MAP_{1}$ decrypts the message and verifies the parameters of $T_{C}$. Further, the nonce $N_{M1}$ is verified by $MAP_{1}$ to check the similarity of the nonce issued by the $MAP_{1}$ in Equation (15). If the verification is successful, then the authentication process is successful between the user and the $MAP_{1}$.
**Step 4:** After successful authentication, when user *C* wants to migrate, it informs to the $MAP_{1}$ to which FMAP the user wants to join. Thereafter, the $MAP_{1}$ generates and sends the encrypted transfer ticket as $E_{CSK_{i}}$ ($\Theta_{C}$) to user *C* and FMAP in Equation (17). Later, user *C* forwards the encrypted transfer ticket $E_{CSK_{i}}$($\Theta_{C}$) to FMAP to authenticate itself.

### 5.2. Proposed Protocol for Handover Authentication Protocol (HAP)

The common secret key (CSK) described in Section 4 is shared among the neighboring MAP’s beforehand the handover process took place to offer privacy. After the completion of mutual trust between the client and HMAP (i.e., $MAP_{1}$), transfer ticket ($\Theta_{C}$) is issued by $MAP_{1}$ to the client and FMAP during the login process as described in Equation (17) of Section 5.1. Later, when the client wants to join the foreign mesh access point (FMAP) during the handover process, the client sends the transfer ticket in an encrypted form as $E_{CSK_{i}}$ ($\Theta_{C}$) to the foreign mesh access point to prove its authenticity as

(18) $$C\to \mathrm{FMAP}:E_{CSK_{i}}\left(\Theta_{C}\right)$$

Step 1. User *C* sends $E_{CSK_{i}}$ ($\Theta_{C}$) to foreign mesh access point (FMAP) as shown in Equation (18). After receiving $E_{CSK_{i}}$ ($\Theta_{C}$), foreign mesh access point (FMAP) tries to decrypt it.

If (successful in decrypting, i.e., $D_{CSK_{i}}$ ($\Theta_{C}$)) then

FMAP verifies the contents of the transfer ticket for successful authentication, i.e., $\Theta_{C}$ sent by HMAP previously during the login process is equal to $\Theta_{C}$ sent by the user during the handover process. If both the contents of $\Theta_{C}$ are similar then the user is authenticated successfully by the foreign mesh access point.

Else

**Figure 3:**
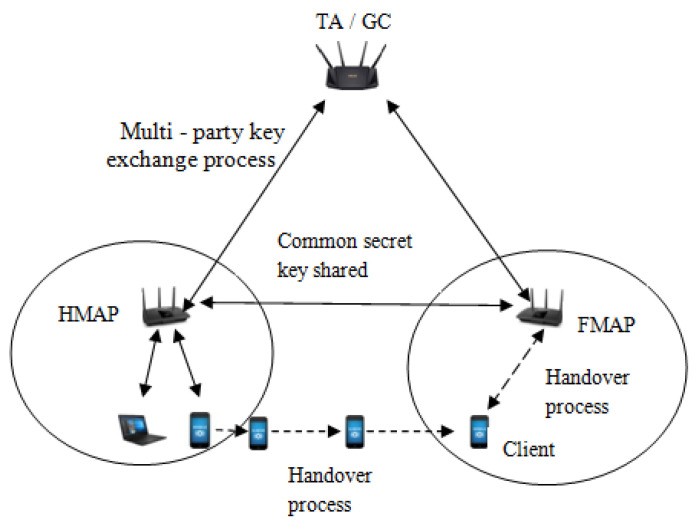
Proposed handover process.

**Figure 4:**
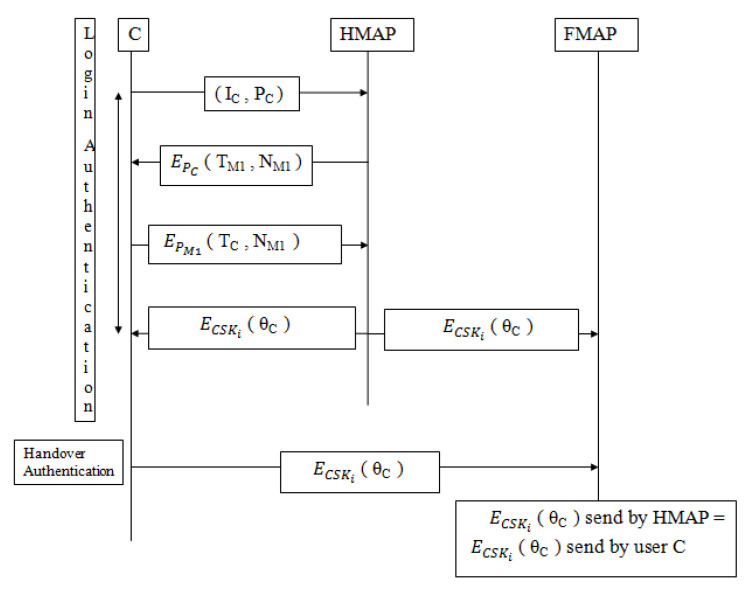
Proposed protocol during LAP and HAP.

If (unsuccessful in decrypting) then

a user fails to authenticate itself to FMAP, as FMAP could not verify the transfer ticket ($\Theta_{C}$) without decrypting it. Finally, FMAP concludes that the transfer ticket ($\Theta_{C}$) was not issued from the corresponding HMAP with whom FMAP had shared the common secret key. Figure 3 shows the handover process of the proposed protocol. Figure 4 shows the proposed login authentication protocol (LAP) and handover authentication protocol (HAP).

## 6. Experimental Results

Implementation and experimental results of our proposed protocol is described in this section. Table 2 shows the experimental model setup, where Network Simulator 3 (NS3) is considered for simulating the proposed protocol as existing protocols have considered the same simulation tool. Other simulation parameters as mentioned in Table 2 is setup based on the existing protocols setup. Table 3 shows the simulation results gained during the login process. Table 4 shows the simulation results gained during the handover process. In both Table 3 and Table 4, *d* represents the average delay transmission within a single hop.

### 6.1. Security Analysis of Proposed Login Authentication Protocol (LAP) and Handover Authentication Protocol (HAP)

In this section we analyze the security of our proposed protocol with respect to the following features:

**Mutual Authentication:** During login operation in Section 5.1, mutual authentication allowed the user and $MAP_{1}$ to verify each others identity. The verification is performed with their respective ticket’s exchanged. $Sig_{A}$ ensures the authentication of the tickets. Later, $MAP_{1}$ encrypts the message through $E_{P_{C}}$ as $E_{P_{C}}$ ($T_{M1},N_{M1}$) shown in Equation (15) and the user encrypts the message through $E_{P_{M1}}$ as $E_{P_{M1}}$ ($T_{C}$, $N_{M1}$) shown in Equation (16). In Section 5.1, encryption of the messages shown in Equations (15) and (16)through public key ensures that only the user C and $MAP_{1}$ can decrypt the message.

**Privacy preservation:** In the Li et al. [12] protocol during LAP and HAP, the information such as $T_{M1}$, $N_{M2}$, $N_{C2}$ and $\Theta_{C}$ are shared in a plaintext format as shown in Equations (5), (8)–(10). As a result, an intruder could easily tamper with the information exchanged during LAP and HAP. Our proposed protocol offers privacy to the exchanged information and prevents from tampering, such as $E_{P_{C}}$ ($T_{M1},N_{M1}$) as shown in Equation (15) and $E_{P_{M1}}$ ($T_{C}$, $N_{M1}$) as shown in Equation (16) during LAP. Privacy of the transfer ticket ($\Theta_{C}$) is also preserved such as $E_{CSK_{i}}$ ($\Theta_{C}$) during LAP in Equation (17) and during HAP in Equation (18); therefore, both mutual authentication and privacy preservation prevents intruders to tamper with the integrity of the exchanged messages and also prevents a replay attack. As a result, the transmitted information could neither be captured by intruders throughout the authentication process, nor could any information be replayed to access the network as a user.

### 6.2. Result Analysis of Proposed Protocol

We considered four performance metrics to compute the overall performance of our proposed protocol. Comparison of proposed protocol with existing protocols is performed in terms of computation and communication cost, login delay, and handover delay.

**Figure 5:**
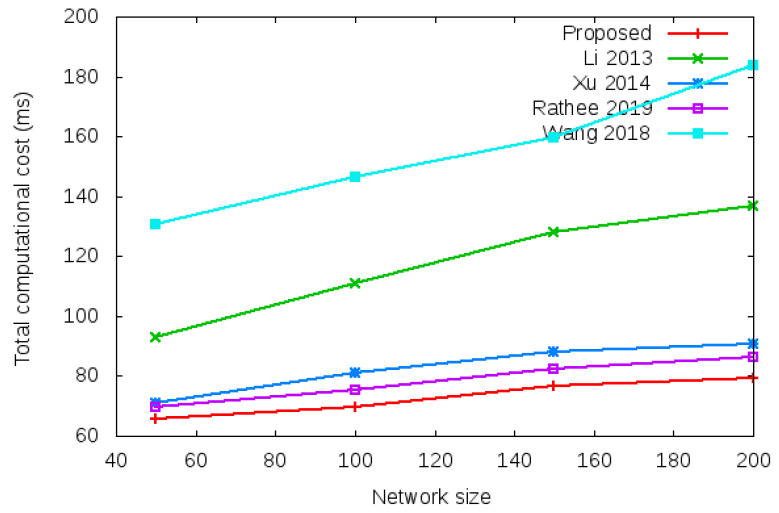
Total computational cost of proposed protocol vs. existing protocols with different network size of 50, 100, 150, and 200 nodes during login authentication process.

**Figure 6:**
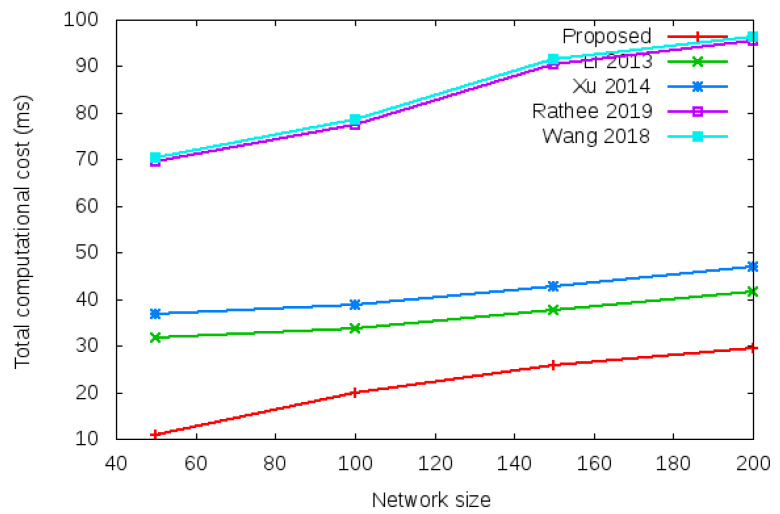
Total computational cost of proposed protocol vs. existing protocols with different network size of 50, 100, 150, and 200 nodes during handover process.

Computational cost is computed as the time required in processing the various security operations given in column 1, row 2, 3, 4, 5, and 6 of Table 3 during login operation and column 1, row 2, 3, 4, 5, and 6 of Table 4 during the handover operation [25,26,27]. Total computation cost comparison during login operation is given in row 7 of Table 3 (i.e., 69.45 vs. 69.54 vs. 69.44 vs. 69.44 vs. 104.16). Total computation cost comparison during handover operation is shown in row 7 of Table 4 (i.e., 0.011 vs. 0.105 vs. 34.78 vs. 69.44 vs. 69.47). Figure 5 shows the total computational cost required during the login authentication process. Figure 6 shows the total computational cost required during the handover authentication process.

**Figure 7:**
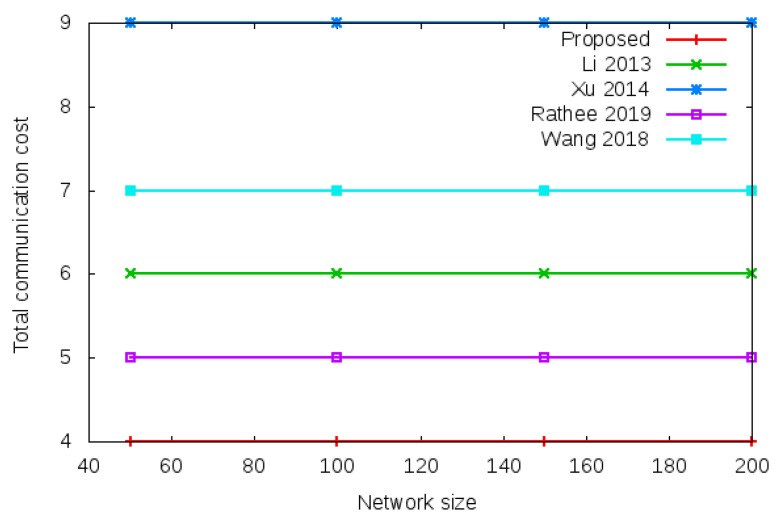
Total communication cost of proposed protocol versus existing protocols with different network size of 50, 100, 150, and 200 nodes during login process. Total communication cost comparison during login operation is given in column 1, row 6 of Table 3 (i.e., 4 vs. 6 vs. 9 vs. 5 vs. 7).

**Figure 8:**
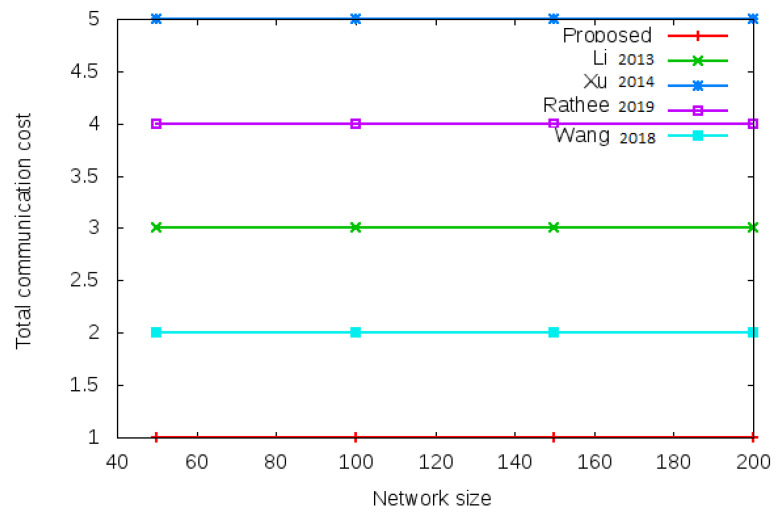
Total communication cost of proposed protocol versus existing protocols with different network size of 50, 100, 150, and 200 nodes during handover process. Total communication cost comparison during handover operation is given in column 1, row 6 of Table 4 (i.e., 1 vs. 3 vs. 5 vs. 4 vs. 2).

Communication cost is the total message exchanged between mesh entities during login operation and handover operation. Figure 7 shows the total communication cost required during the login authentication process. Total communication cost is the number of messages exchanged during the login operation given in column 1, row 6 of Table 3 (i.e., 4 vs.6 vs. 9 vs. 5 vs. 7). Figure 8 shows the total communication cost required during the handover authentication process. Total communication cost is the number of messages exchanged during handover operation given in column 1, row 6 of Table 4 (i.e., 1 vs. 3 vs. 5 vs. 4 vs. 2).

**Figure 9:**
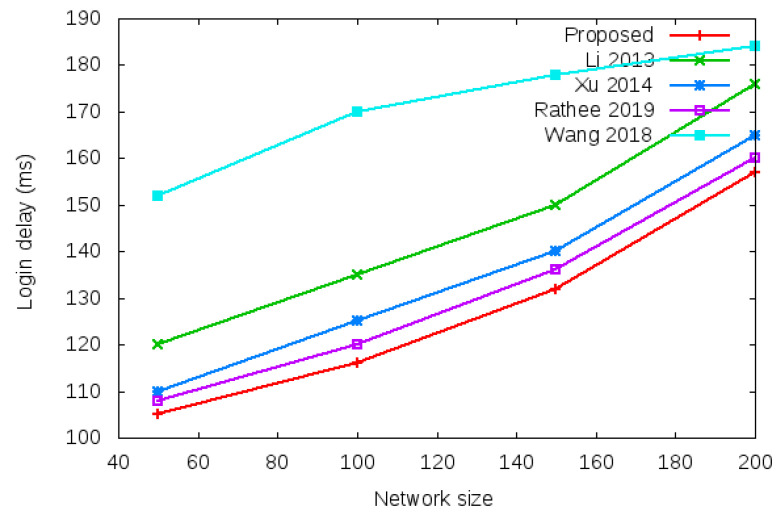
Login delay of the proposed protocol versus existing protocols with a different network size of 50, 100, 150, and 200 nodes based on total computational cost and total communication cost during the login authentication process.

**Figure 10:**
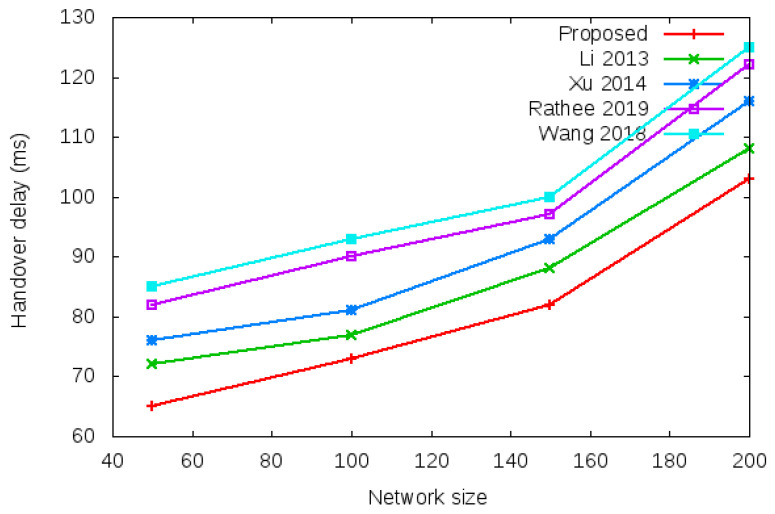
Handover delay of proposed protocol versus existing protocols with a different network size of 50, 100, 150, and 200 nodes based on total computational cost and total communication cost during the handover authentication process.

Login delay and handover delay are the time utilized during sending an authentication request and receiving the acceptance confirmation among mesh entities. The time utilized is the addition of computation cost and communication cost shown in the bottom row of Table 3 and Table 4. Symbol *d* in the bottom row of Table 3 and Table 4 denotes average delay transmission within a single hop. Figure 9 shows the login delay required during the login authentication process. Login delay is the time utilized during sending an authentication request and receiving the acceptance confirmation among mesh entities during the login process. The simulation result is shown in the bottom row of Table 3. Figure 10 shows the handover delay required during the handover authentication process. Handover delay is the time utilized during sending an authentication request and receiving the acceptance confirmation among mesh entities during the handover process. The simulation result is shown in the bottom row of Table 4.

**Figure 11:**
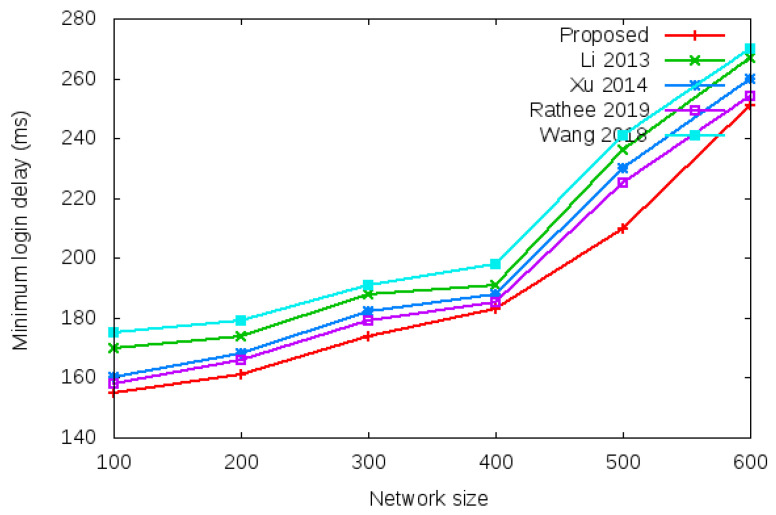
Minimum login authentication delay.

Table 5 and Figure 11 show the results of minimum login authentication delay with the network size of 100 to 600 mobile clients.

**Figure 12:**
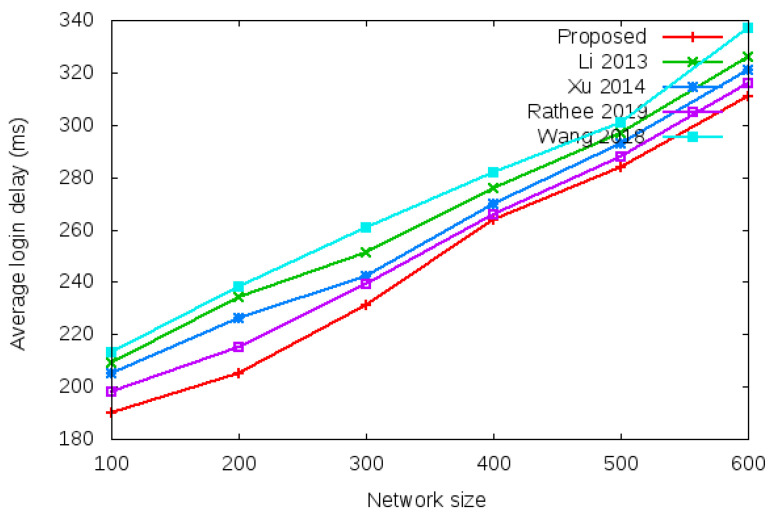
Average login authentication delay.

Table 6 and Figure 12 show the results of average login authentication delay with the network size of 100 to 600 mobile clients.

**Figure 13:**
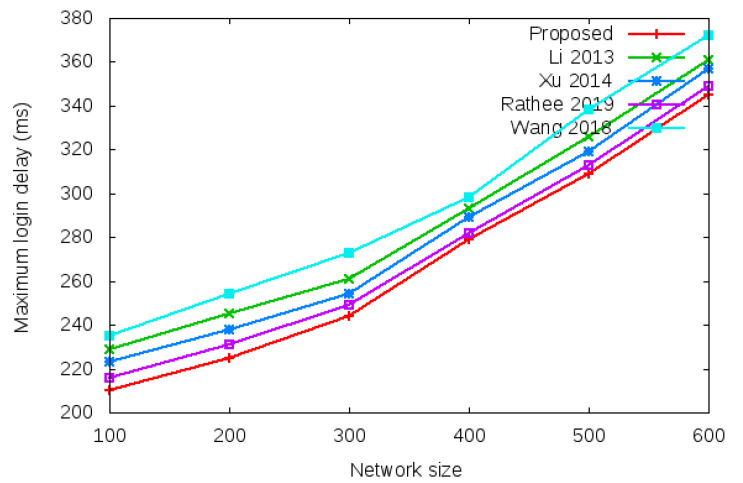
Maximum login authentication delay.

Table 7 and Figure 13 show the results of maximum login authentication delay with the network size of 100 to 600 mobile clients.

**Figure 14:**
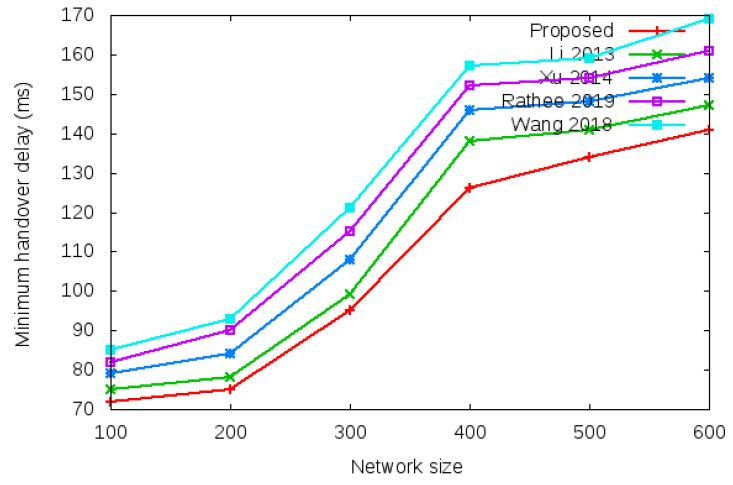
Minimum handover authentication delay.

Table 8 and Figure 14 show the results of minimum handover authentication delay with the network size of 100 to 600 mobile clients.

**Figure 15:**
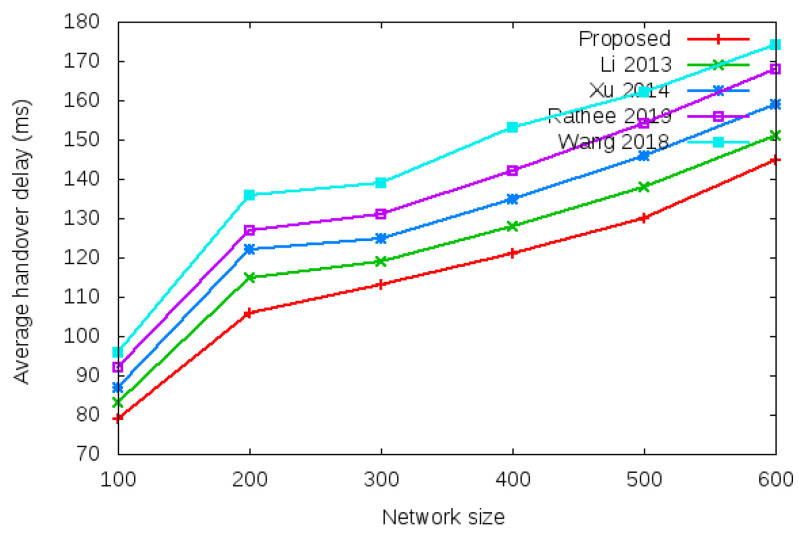
Average handover authentication delay.

Table 9 and Figure 15 show the results of average handover authentication delay with the network size of 100 to 600 mobile clients.

**Figure 16:**
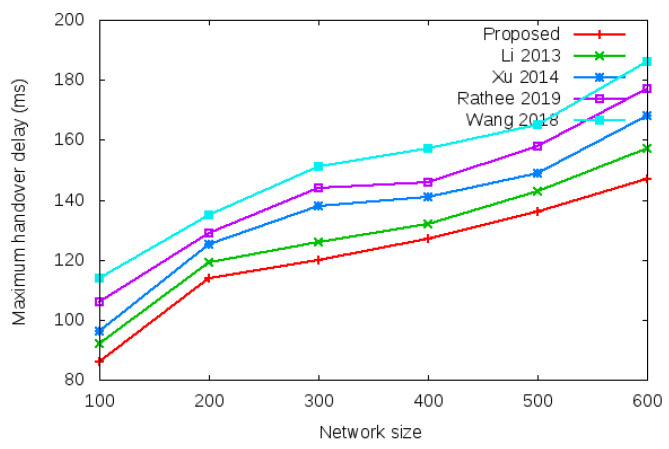
Maximum handover authentication delay.

Table 10 and Figure 16 show the results of maximum handover authentication delay with the network size of 100 to 600 mobile clients.

## 7. Conclusions

Multi-party key exchange protocol preserves the privacy of the exchanged information shared during the login authentication process (LAP) and handover authentication process (HAP) to offer secure communication. The experimental results show that the proposed protocol achieves minimum authentication delay compared to existing protocols in terms of computation cost and communication cost. Through security analysis, it also proves that the proposed protocol offers a higher security level during the login authentication process (LAP) and handover authentication process (HAP) where no intruders can tamper with the exchanged information. In the future, the proposed protocol can be further extended to gain more efficiency and security during the login authentication process (LAP) and handover authentication process (HAP) for wireless mesh networks (WMN).

## Figures and Tables

**Table 1:** Comparison of protocols during handover operation.

Protocol	$\Theta_{C}$ Issued by	Privacy	Authent. Process	Compt. Cost	Commt. Cost	Authent. Delay
Kassab et al. [13]	AS	Yes	Multi-hop	High	High	High
Li et al. [14]	AS	Yes	Multi-hop	High	High	High
Li et al. [15]	AS	Yes	Multi-hop	High	High	High
He et al. [16]	AS	Yes	Multi-hop	High	Low	Low
Xu et al. [17]	AS	Yes	Multi-hop	High	High	High
Rathee et al. [18]	AS	No	Multi-hop	High	High	High
Wang et al. [19]	AS	Yes	Multi-hop	High	High	High
Rekik et al. [20]	AS	Yes	Multi-hop	High	High	High
Tsai et al. [21]	AS	Yes	Multi-hop	High	High	High
Fu et al. [22]	AS	Yes	Multi-hop	High	High	High
Zhu et al. [23]	AS	Yes	Multi-hop	High	High	High
Yang et al. [24]	AS	Yes	Multi-hop	High	Low	Low
Li et al. [12]	MAP	No	One-hop	Low	High	Low
Proposed	MAP	Yes	One-hop	Low	Low	Low

**Table 2:** Experimental model setup.

Notation	Description
Platform	NS3
Traffic	CBR/UDP
Routing Protocol	AODV
Simulation Area	1000 × 1000 m
MAC protocol	IEEE 802.11
Total MAP	4
Placement of nodes	Randomly
Network size	50, 100, 150, 200
MAPs Transmission range	250 m
Clients Transmission range	100 m

**Table 3:** Simulation results during login process.

Operation	Algorithm	Time	Proposed	Li et al. [12]	Xu et al. [17]	Rathee et al. [18]	Wang et al. [19]
$E_{pub_{x}}$ (m)	RSA	1.42	2	2	2	2	3
$D_{prv_{x}}$ (m)	RSA	33.3	2	2	2	2	3
$E_{CSK}$ (m)	AES	0.016	1	0	0	0	0
$D_{CSK}$ (m)	AES	0.011	0	0	0	0	0
MAC	HMAC	0.015	0	2	0	0	0
Comput.cost (ms)	-	-	69.45	69.54	69.44	69.44	104.16
No. of messages	-	-	4	6	9	5	7
Login delay (ms)	-	-	69.45 + 4d	69.54 + 6d	69.44 + 9d	69.44 + 5d	104.16 + 7d

**Table 4:** Simulation results during handover process.

Operation	Algorithm	Time	Proposed	Li et al. [12]	Xu et al. [17]	Rathee et al. [18]	Wang et al. [19]
$E_{pub_{x}}$ (m)	RSA	1.42	0	0	1	2	2
$D_{prv_{x}}$ (m)	RSA	33.3	0	0	1	2	2
$E_{CSK}$ (m)	AES	0.016	0	0	0	0	0
$D_{CSK}$ (m)	AES	0.011	1	0	0	0	0
MAC	HMAC	0.015	0	7	4	0	2
Comput.cost (ms)	-	-	0.011	0.105	34.78	69.44	69.47
No. of messages	-	-	1	3	5	4	2
Handover delay (ms)	-	-	0.011 + 1d	0.105 + 3d	34.78 + 5d	69.44 + 4d	69.47 + 2d

**Table 5:** Comparison on minimum login authentication delay.

Number of Mobile Clients	Proposed	Li et al. [12]	Xu et al. [17]	Rathee et al. [18]	Wang et al. [19]
100	155	170	160	158	175
200	161	174	168	166	179
300	174	188	182	179	191
400	183	191	188	185	198
500	210	236	230	225	241
600	251	267	260	254	270

**Table 6:** Comparison on average login authentication delay.

Number of Mobile Clients	Proposed	Li et al. [12]	Xu et al. [17]	Rathee et al. [18]	Wang et al. [19]
100	190	209	205	198	213
200	205	234	226	215	238
300	231	251	242	239	261
400	264	276	270	266	282
500	284	297	293	288	301
600	311	326	321	316	337

**Table 7:** Comparison on maximum login authentication delay.

Number of Mobile Clients	Proposed	Li et al. [12]	Xu et al. [17]	Rathee et al. [18]	Wang et al. [19]
100	210	229	223	216	235
200	225	245	238	231	254
300	244	261	254	249	273
400	279	293	289	282	298
500	309	326	319	313	338
600	345	361	357	349	372

**Table 8:** Comparison on minimum handover authentication delay.

Number of Mobile Clients	Proposed	Li et al. [12]	Xu et al. [17]	Rathee et al. [18]	Wang et al. [19]
100	72	75	79	82	85
200	75	78	84	90	93
300	95	99	108	115	121
400	126	138	146	152	157
500	134	141	148	154	159
600	141	147	154	161	169

**Table 9:** Comparison on average handover authentication delay.

Number of Mobile Clients	Proposed	Li et al. [12]	Xu et al. [17]	Rathee et al. [18]	Wang et al. [19]
100	79	83	87	92	96
200	106	115	122	127	136
300	113	119	125	131	139
400	121	128	135	142	153
500	130	138	146	154	162
600	145	151	159	168	174

**Table 10:** Comparison on maximum handover authentication delay.

Number of Mobile Clients	Proposed	Li et al. [12]	Xu et al. [17]	Rathee et al. [18]	Wang et al. [19]
100	86	92	96	106	114
200	115	119	125	129	135
300	120	126	138	144	151
400	127	132	141	146	157
500	136	143	149	158	165
600	147	157	168	177	186

## Data Availability

The data presented in this study are available on request from the corresponding author.

## Abbreviations

The following abbreviations are used in this manuscript:

g	generator
mod *p*	modulo prime
HMAP	Home mesh access point
FMAP	Foreign mesh access point
TA/GC	Ticket agent/Group controller
AS	Authentication server
pub	Public key of x
prv	Private key of x
CSK	Common secret key
ID	mesh entities identity
LAP	Login authentication protocol
HAP	Handover authentication protocol
MAC	Message Authentication Code
$E_{CSK}$ (m)	Encryption of message with CSK
$D_{CSK}$ (m)	Decryption of message with CSK
$T_{C}$	User Ticket
$T_{M}$	MAP Ticket
$\Theta_{C}$	Transfer ticket
$Sig_{A}$	Digital Signature of AS
MC	Mesh client
MR	Mesh router
GW	Gateway
PMK	Pairwise Master Key
